# SurvivalGWAS_Power: a user friendly tool for power calculations in pharmacogenetic studies with “time to event” outcomes

**DOI:** 10.1186/s12859-016-1407-9

**Published:** 2016-12-08

**Authors:** Hamzah Syed, Andrea L. Jorgensen, Andrew P. Morris

**Affiliations:** 1 Department of Biostatistics, University of Liverpool, Liverpool, UK; 2 Department of Molecular and Clinical Pharmacology, University of Liverpool, Liverpool, UK

**Keywords:** Pharmacogenetics, Power calculation, Time to event, Cox proportional hazards, Weibull regression, Simulation, Censoring, SNP-treatment interaction

## Abstract

**Background:**

Power calculators are currently available for the design of genetic association studies of binary phenotypes and quantitative traits, but not for “time to event” outcomes, which are of particular relevance in pharmacogenetics. With the rapid emergence of pharmacogenetic association studies of single nucleotide polymorphisms (SNPs), and the complexity of clinical outcomes they consider, there is a need for software to perform power calculations of time to event data over a range of design scenarios and analytical methodologies.

**Results:**

We have developed the user friendly software tool SurvivalGWAS_Power to perform power calculations for time to event outcomes over a range of study designs and different analytical approaches. The software calculates the power to detect SNP association with a time to event outcome over a range of study design scenarios. The software enables analyses under a Cox proportional hazards model or Weibull regression model, and can account for treatment and SNP-treatment interaction effects. Simulated data sets can also be generated by SurvivalGWAS_Power to enable analyses with methods that are not currently supported by the power calculator, thereby increasing the flexibility of the software.

**Conclusions:**

SurvivalGWAS_Power addresses the need for flexible and user-friendly software for power calculations for genetic association studies of time to event outcomes, with particular design features of relevance in pharmacogenetics.

**Electronic supplementary material:**

The online version of this article (doi:10.1186/s12859-016-1407-9) contains supplementary material, which is available to authorized users.

## Background

Power calculations are an essential component of study design, and are readily available for genome-wide association studies (GWAS) of binary phenotypes and quantitative traits [1]. However, as yet, software is not available to determine adequate sample size for GWAS of “time to event” outcomes. Such outcomes are of particular relevance in the emerging field of pharmacogenetics, where the event could be death, disease remission, or the occurrence of an adverse drug reaction, for example, after treatment intervention. The most appropriate approach to the analysis of time to event data is through survival modelling, which can allow for censoring because the event has not occurred, or due to patient drop-out, before the end of the study. Software is thus required to enable power calculations for GWAS of time to event outcomes, for alternative analytical models, particularly for pharmacogenetic studies, where the impact of alternative treatments, and potentially their interaction with single nucleotide polymorphisms (SNPs), is often of relevance in the study design.

The objective of GWAS is to identify SNPs that are associated with outcome, typically at a stringent level of genome-wide significance (defined as *p* < 5 × 10^−8^) [2]. SNPs are variations in the DNA sequence and are encoded by genotypes. Genotypes can be represented as AA, AB and BB, where A and B are the two alternative alleles at the SNP. Association of a SNP with outcome can then be assessed within a generalised linear modelling framework, accounting for relevant confounding variables as covariates. It is typical to assume an “additive” genetic model, whereby the effect of the heterozygous AB genotype is intermediate between homozygous AA and BB genotypes. Under this additive model, genotypes are coded according to the number of B alleles carried, such that AA = 0, AB = 1 and BB = 2.

Currently, software to determine power for GWAS of binary phenotypes and quantitative traits is available. These include the freely available ‘Genetic Power Calculator’, developed by Purcell et al. [1], which is a web based platform, and ‘genomeSIM’ developed by Dudek et al. [3] for the simulation of large-scale genomic data in population based case-control samples. However, as yet, software for power calculations for GWAS of time to event outcomes is not available. In response to this analytical bottleneck, we have developed the user friendly tool, SurvivalGWAS_Power, which is the first program to implement both data generation and power calculations for GWAS of time to event outcomes. The software is of particular relevance to pharmacogenetic studies, where the design will likely include alternative treatment interventions, and analyses are likely to consider SNP-treatment interaction effects.

## Implementation

SurvivalGWAS_Power was built using C# and developed as a Windows application, utilising pre-designed frameworks Math.NET and Accord.NET [4], for the generation of pharmacogenetic data and statistical analyses, respectively. SurvivalGWAS_Power requires specification of genetic parameters, such as the magnitude of the SNP effect on the outcome and the minor allele frequency (MAF). The varied collection of design scenarios includes adding a recruitment period, SNP-treatment interactions, and/or different censoring options (for example, withdrawal due to an adverse treatment event). We created these pharmacogenomic study designs after a thorough examination of published studies in the literature [5], including Charland et al. [6], Depta et al. [7], Wiese et al. [8], and Absenger et al. [9]. The power calculation is performed by simulating multiple datasets based on the user specified parameter settings and study design options, specifically testing for SNP associations (and SNP-treatment interactions, if required) with the time to event outcome across all simulated datasets. For GWAS, the usual threshold for “genome-wide significance” is *p* < 5 × 10^−8^. However, the software is equally applicable to power calculations for individual SNPs, for which a nominal significance threshold of *p* < 0.05 is appropriate.

### User interface

The main window consists of two panels, the first for design, analysis and parameter inputs, and the second for all output. The menu bar has a “Save Sample Data” option, as well as another option to store all the datasets from every simulation run. This is useful for those who want to test power for methods not supported by the program. The data are saved as a text file, in R statistical software friendly readable format. The interface has been designed to be user friendly; there are various help buttons to navigate the user through the program in the form of tooltips, and an example of a commonly used pharmacogenetic study design is available as a guide. The inputs are split into two sections: (i) data generation inputs; and (ii) statistical analysis inputs. The user defined parameter inputs are submitted in text boxes.

### Data simulation

For each replicate of data, a SNP genotype (coded as 0, 1 or 2) is generated for each individual from a binomial distribution dependent on the MAF and assuming Hardy-Weinberg equilibrium. The user is given the option of incorporating an active treatment against a placebo. Treatment allocation is also simulated using a binomial distribution.

Time to event for each individual is then simulated on the basis of specified model parameters from a Weibull distribution, which allows for the possibility of a deviation from a proportional hazards assumption. The value of the shape parameter, *a*, of the Weibull distribution is specified by the user. A value of *a* < 1 indicates that the failure rate decreases over time. A value of *a* = 1 indicates that the failure rate is constant over time, resulting in proportional hazards. A value of *a* < 1 indicates that the failure rate increases with time. The scale parameter of the Weibull distribution is parameterized to incorporate SNP, treatment, and SNP-treatment interaction effects in generating time to event for each individual. Specifically, the scale parameter for the *i*th individual is given by, ${b}_i={d}_0{e}^{\beta_s{S}_i+{\beta}_x{x}_i+{\beta}_{\gamma }{S}_i{x}_i}$, where *x*_*i*_ is the treatment covariate (coded as 0/1 for placebo/active), *S*_*i*_ is the SNP genotype coded under an additive model for the minor allele. The value of the “baseline” scale parameter *d*_0_, is specified by the user. The parameters *β*_*s*_ and *β*_*x*_ are the effect on log-hazard of the minor allele at the SNP, and the treatment effect, respectively, and *β*_*γ*_ is the interaction effect between the SNP and treatment. The values of each of these parameters are also specified by the user.

The simulated observed time to event outcome is generated for the following possible study design scenarios, each of which include the option of incorporating treatment and SNP-treatment interaction effects. In all scenarios, the simulated time to event of the *i*th individual is denoted *t*_*i*_, and the observed event time (after censoring) is denoted *δ*_*i*_.

- Scenario 1-End of study censoring.
  This scenario is designed based on a user specified and fixed end of study time, Z. If the event occurs before the end of the study and due to this scenario not including censoring before time Z, the observed event time for the *i*th individual is *δ*_*i*_ = *t*_*i*_; otherwise *δ*_*i*_ = *Z*.
- Scenario 2-Censoring during the study period and at end of study.
  The censoring time of the *i*th individual, *c*_*i*_, is simulated from a Weibull distribution with a user defined scale parameter and a fixed shape parameter of 1. Small values of the scale parameter will generate more censored observations. If censoring occurs before the end of the study, the individual is assumed to have dropped out at that time, thus *δ*_*i*_ = *c*_*i*_. If censoring occurs after the end of the study, yet the event occurred before the end of the study, then the observed event time for the *i*th individual is *δ*_*i*_ = *t*_*i*_ − *r*_*i*_; otherwise *δ*_*i*_ = *Z* − *r*_*i*_.
- Scenario 3-Recruitment period and end of study censoring.
  The recruitment time, *r*_*i*_, is simulated from a discrete uniform distribution between 0 and a specified end time. There is no censoring before the end of the study and if the event occurs before the end of the study the observed event time for the *i*th individual is *δ*_*i*_ = *t*_*i*_ − *r*_*i*_; otherwise *δ*_*i*_ = *Z* − *r*_*i*_.
- Scenario 4-Censoring during the study period and at the end of study with a recruitment period.
  The censoring time of the *i*th individual, *c*_*i*_, is simulated from a Weibull distribution with a user defined scale parameter and a fixed shape parameter of 1. If censoring does not occur before the end of study, and an event has occurred, an individual will have observed time *δ*_*i*_ = *t*_*i*_–*r*_*i*_, unless censored at the end of study, then *δ*_*i*_ = *Z* − *r*_*i*_. If censoring does occur during the study period, then *δ*_*i*_ = *c*_*i*_ − *r*_*i*_.

### Analysis

There are two options to compare when running the analysis; (i) a Cox proportional hazards model and (ii) a Weibull regression model. Users can select between running their choice of analysis model by fitting: (i) the SNP alone; (ii) the SNP and treatment; or (iii) the SNP, treatment and SNP-treatment interaction.

The Cox proportional hazards regression model is the most extensively used analysis of time to event outcomes. It is a semi-parametric model that assumes that the hazard functions of individuals are proportional over time. The framework Accord.Net has a built in Cox proportional hazards function, which calculates the partial likelihood and obtains parameter estimates and Wald test *p*-value.

The Weibull regression model is parametric with completely specified hazard and survivor functions, and is beneficial in scenarios where the hazard is not proportional or has an accelerated failure time feature. We obtain maximum likelihood estimates of model parameters using an iterative Newton-Raphson method, the full derivation of which is presented in Additional file 1.

### Validation

**Fig. 1:**
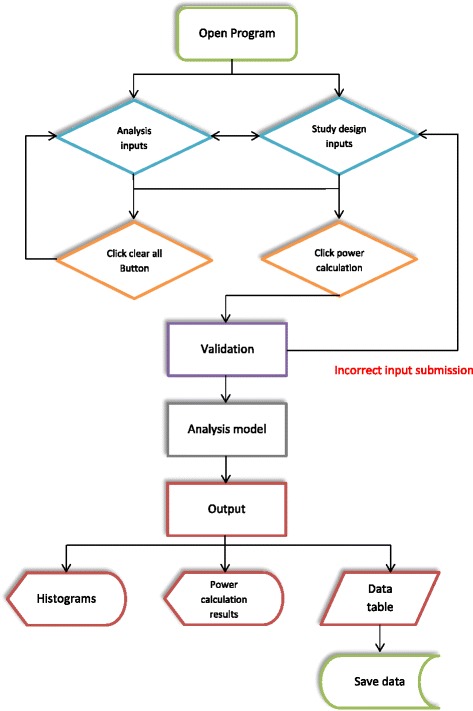
Workflow diagram of software functionality

The user interface implements a validation system to track user errors at input. As the user inputs values into parameter textboxes, the error provider will check that the entered values are valid. Before the power calculation begins, the error provider will check that all required information has been entered for a selected scenario. For example, the user cannot select treatment as an analysis covariate if a treatment effect has not been included in the simulation model. The workflow is presented in Fig. 1.

### Output

The output comprises of a sample dataset, a table of the analysis output for each simulation run and two histograms of parameters across simulations: (i) coefficient values for the SNP effect from the regression model; and (ii) ‐ *log*_10_ Wald *p*-values for the SNP effect. All histograms can be saved by right clicking the graph and selecting “save as image”. Power, at the specified significance threshold, *φ*, is approximated by the proportion of replicates for which *p* < *φ* for the SNP effect on outcome. Power, at the same significance threshold, is also calculated for the SNP-treatment interaction effect, if this term is included in the analysis model.

## Results

SurvivalGWAS_Power can simulate a large number of datasets to enable efficient estimation of power based on specified model parameters and design scenarios. We present the results of example power calculations for two similar scenarios to demonstrate the utility of the software.

**Fig. 2:**
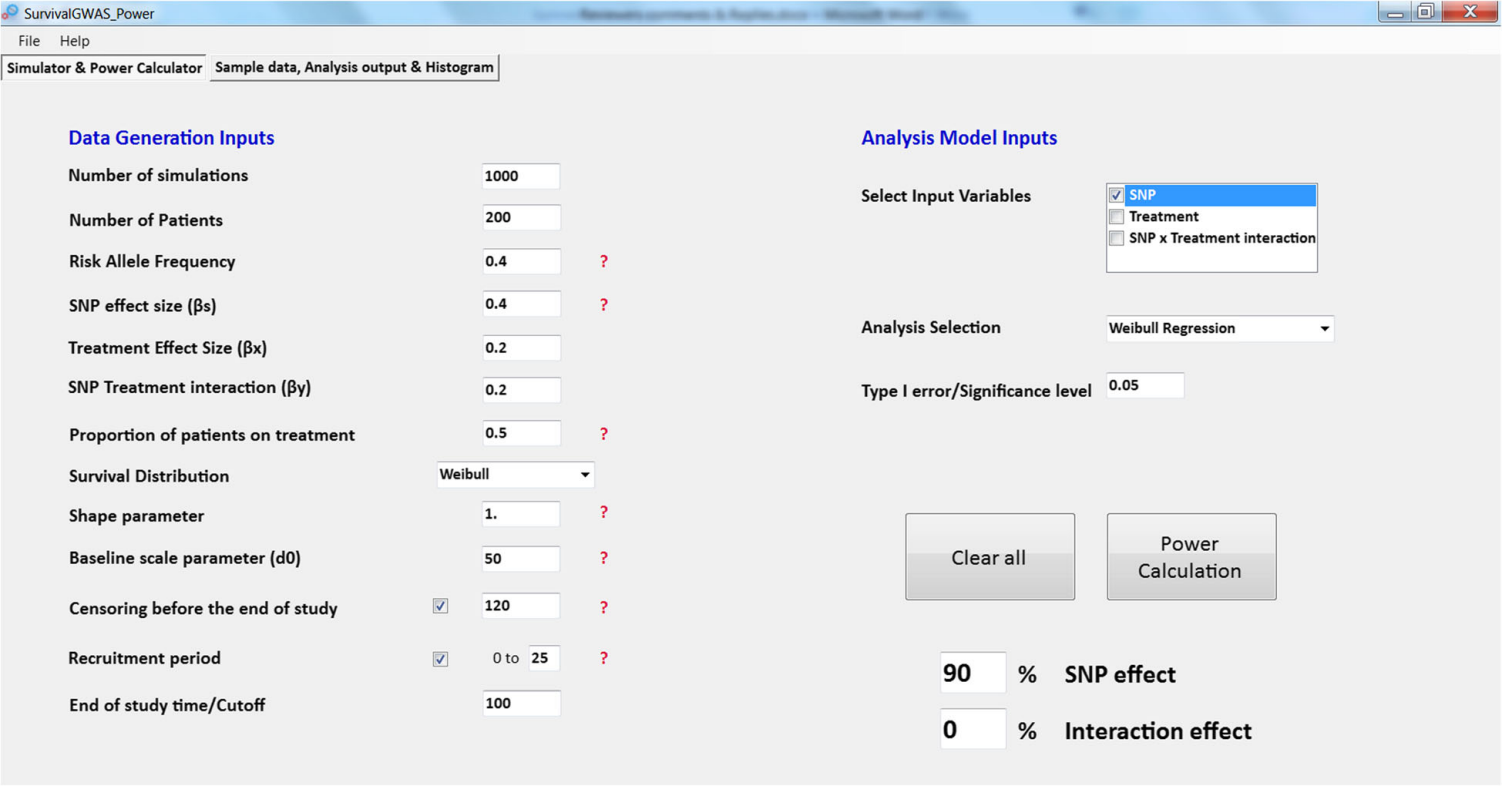
Front end of SurvivalGWAS_Power. Legend: Input parameter tab for simulations and analysis. Depicting a scenario with only a SNP effect analysed, censoring and a recruitment period

Figure 2 presents the input parameter tab of the software. The example demonstrates a scenario with censoring during the study period and at the end of the study, and including a recruitment period. Here we have considered SNP and treatment main effects and a SNP-treatment interaction effect in simulating event times. However, in the analysis, we have only considered the SNP main effect. Specifically, a Weibull regression model is implemented for analysis to test the SNP association (i.e. the null hypothesis H_0_ : *β*_*s*_ = 0 against the alternative H_A_ : *β*_*s*_ ≠ 0), for which power is estimated to be 90% at a significance threshold of *p* < 0.05.

**Fig. 3:**
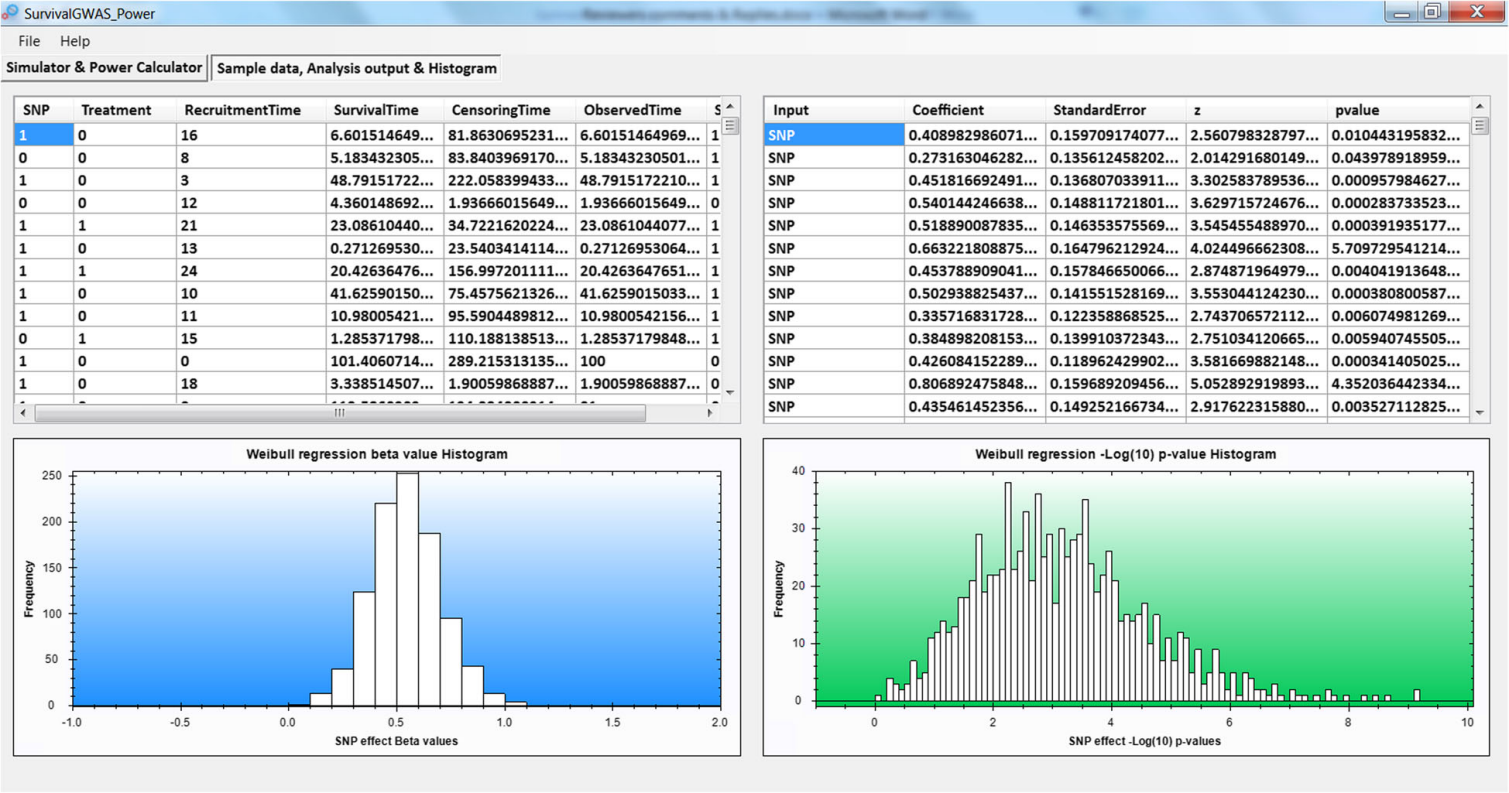
Output tab of SurvivalGWAS_Power. Legend: Output from example power calculation. (*Top left*) sample dataset, (*Top right*) Parameter estimates of the SNP effect from each simulation run, (*Bottom left*) histogram of SNP coefficient beta effects across simulations & (*Bottom right*) histogram of ‐ *log*_10_ *p*-values for the SNP effect across simulations

Figure 3 shows the additional output from the analysis. This is the output from the setup shown in Fig. 2. The left histogram shows the distribution of estimated SNP effect sizes across simulations, which in this example are centred around 0.5, and not the true effect size of 0.4. This bias occurs as the data are simulated with treatment and SNP-treatment interaction effects, but the analysis model does not take these into account.

**Fig. 4:**
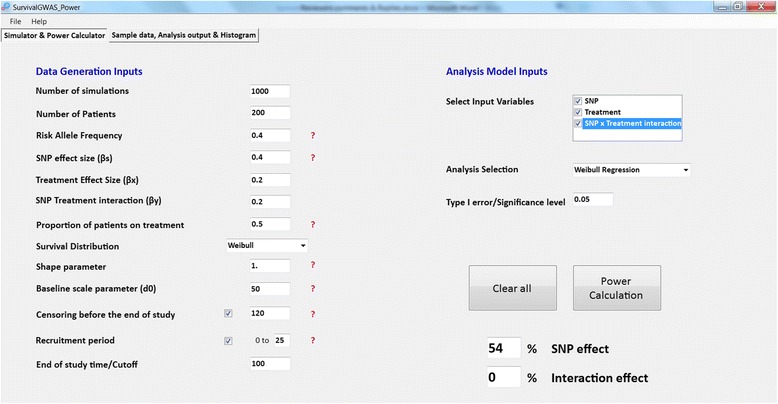
Front end of SurvivalGWAS_Power. Legend: Input parameter tab for simulations and analysis. Depicting a scenario with a SNP effect, treatment and interaction effect analysed, censoring and a recruitment period

Figure 4 presents the input parameter tab of the software with an alternative scenario to Fig. 2. We have used the same simulation model as in the previous example, including censoring during the study period and at end of study with a recruitment period. Here we have also considered SNP and treatment main effects and a SNP-treatment interaction effect in simulating event times. However, in the analysis, we have considered the SNP main effect along with treatment and an interaction effect. A Weibull regression model is implemented for analysis to test: (i) the null hypothesis H_0_ : *β*_*s*_ = 0 against the alternative H_A_ : *β*_*s*_ ≠ 0, for which power is estimated to be 54% at a significance threshold of *p* < 0.05; and (ii) the null hypothesis H_0_ : *β*_*γ*_ = 0 against the alternative H_A_ : *β*_*γ*_ ≠ 0, for which power is estimated to be 0% at a significance threshold of *p* < 0.05.

**Fig. 5:**
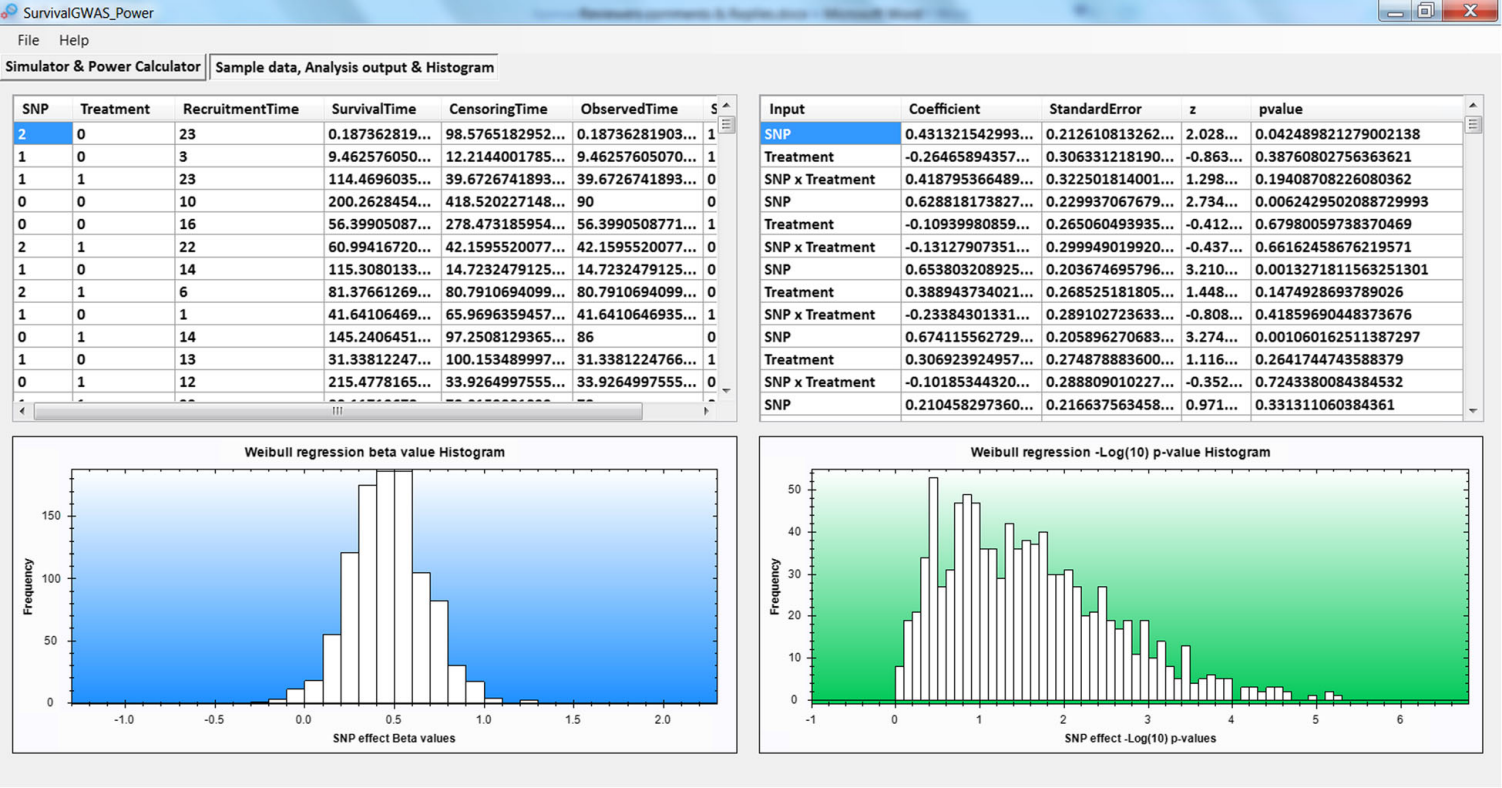
Output tab of SurvivalGWAS_Power. Legend: Output from example power calculation. (*Top left*) sample dataset, (*Top right*) Parameter estimates of the SNP effect from each simulation run, (*Bottom left*) histogram of SNP coefficient beta effects across simulations & (*Bottom right*) histogram of ‐ *log*_10_ *p*-values for the SNP effect across simulations

Figure 5 shows the additional output from the analysis. This is the output from the setup shown in Fig. 4. The left histogram shows the distribution of estimated SNP effect sizes across simulations, which in this example are centred around 0.4. Unlike the previous example, the effect estimates are now unbiased because the correct analysis model is fitted by accounting for treatment and SNP-treatment interaction effects.

### Performance

**Fig. 6:**
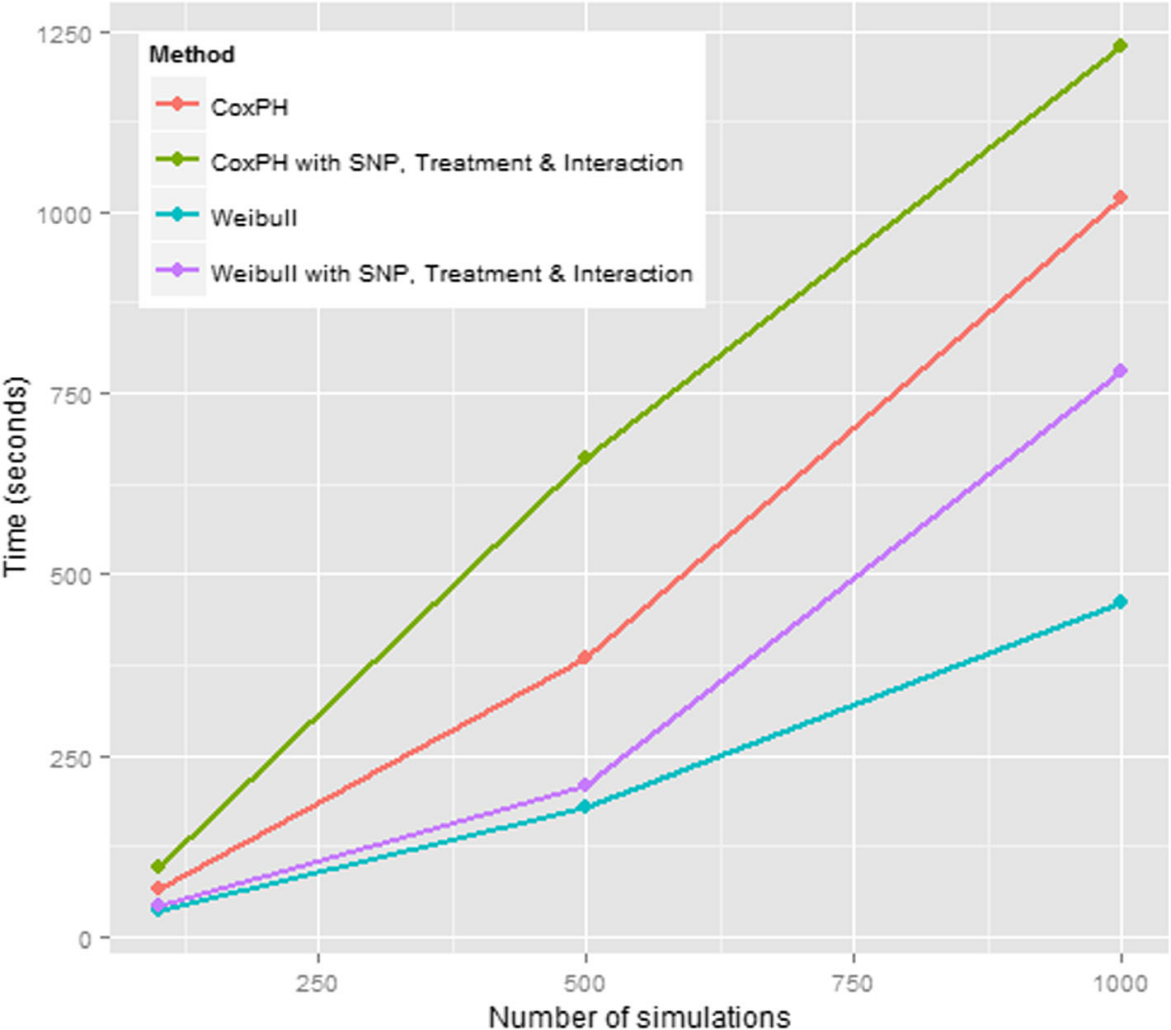
Performance graph—Sample size of 1000 with censoring. Legend: Performance of SurvivalGWAS_Power comparing alternative regression models. Sample size of 1000 used for each simulation

Figure 6 presents run times of SurvivalGWAS_Power under two different analyses as a function of the number of simulations: (i) SNP effect only; and (ii) SNP effect, treatment effect and SNP-treatment interaction. Results are presented for a Cox proportional hazards model and Weibull regression model, for a sample size of 1000 individuals and a scenario with censoring but no recruitment period.

## Conclusions

SurvivalGWAS_Power effectively and efficiently estimates the power of the Cox proportional hazards model and Weibull regression model under a variety of pharmacogenetic settings. Specifically, we allow for testing of SNP main effects (i.e. testing the null hypothesis *H*_0_ : *β*_*s*_ = 0 against the alternative H_A_ : *β*_*s*_ ≠ 0) and SNP-treatment interaction effects (i.e. testing the null hypothesis H_0_ : *β*_*γ*_ = 0 against the alternative H_A_ : *β*_*s*_ ≠ 0).

The software offers the option for users to simulate data and use other programs such as R for analysis. For example, Uno et al. [10] have demonstrated that, where the proportional hazards assumption is invalid, the use of the Cox proportional hazards method will produce a loss in power to detect associations. They propose using alternative robust measures for the difference between survival curves instead of parametric models. The flexibility of our software enables generation of time to event data under models with non-proportional hazards that can be exported for association testing with methods not supported by our power calculator.

SurvivalGWAS_Power is important as it is the first genetic data simulator with time to event outcomes, and the first to enable estimation of power for multiple pharmacogenetic designs and analysis methods. However, the software is not limited to pharmacogenetic designs: for example, the treatment can be used to represent any binary covariate. This adds flexibility to the software for application to general GWAS of time to event outcomes.

In summary, this application offers a much needed user-friendly and flexible software tool for power calculations for time to event outcomes in pharmacogenetic association study designs.

## Additional file

Additional file 1: Details of Weibull regression model. (DOCX 14 kb)

## Abbreviations

GWAS: Genome-wide association studies
MAF: Minor allele frequency
SNPs: Single nucleotide polymorphisms
