# Supplementary material for: SurvivalGWAS_Power: a user friendly tool for power calculations in pharmacogenetic studies with “time to event” outcomes
Source: BMC Bioinformatics. 2016 Dec 8;17:523. doi: 10.1186/s12859-016-1407-9 (PMC5146816; doi:10.1186/s12859-016-1407-9)
Supplement: Additional file 1: — Details of Weibull regression model. (DOCX 14 kb) [file 12859_2016_1407_MOESM1_ESM.docx]

**Supplementary Information**

We model time to event data within a Weibull regression framework. We denote the time to event for the $i$th patient by$t_{i}$, and their treatment by covariate$x_{i}$. We also denote their genotype at a SNP of interest by$S_{i}$, coded under an additive dosage model (directly typed or imputed) for the minor allele. Within this framework, the time to event for the $i$th patient is modelled by a Weibull distribution with shape parameter, $a$, and scale parameter ${b_{i}=d}_{0}e^{\beta_{s}S_{i}{+\beta}_{x}x_{i}+\beta_{\gamma}S_{i}x_{i}}$. The parameters $\beta_{s}$and $\beta_{x}$ are the effect on hazard of the minor allele at the SNP, and the treatment effect, respectively, and$\beta_{\gamma}$ is the interaction effect between the SNP and treatment, whilst $d_{0}$ is the “baseline” scale parameter.

The likelihood of the observed time to event data under the Weibull model is then given by

$L\left( \mathbf{t},\mathbf{c} | {a,d}_{0},\beta_{s},\beta_{x},\beta_{\gamma} \right)=\prod_{i} {f(t_{i}|{a,d}_{0},\beta_{s},\beta_{x},\beta_{\gamma})}^{c_{i}}{S(t_{i}|{a,d}_{0},\beta_{s},\beta_{x},\beta_{\gamma})}^{{1-c}_{i}}$,

Where $c_{i}=0$ if the $i$th patient is censored, and 1 otherwise, and

$$f\left( t_{i} | {a,d}_{0},\beta_{s},\beta_{x},\beta_{\gamma} \right)=ab_{i}t_{i}^{a-1}\exp\left( -b_{i}t_{i}^{a} \right)$$

$S\left( t_{i} | {a,d}_{0},\beta_{s},\beta_{x},\beta_{\gamma} \right)=\exp\left( -b_{i}t_{i}^{a} \right)$.

We obtain maximum likelihood estimates for the set of model parameters $\boldsymbol{\theta}=\{a,d_{0},\boldsymbol{\beta}\}$ using a Newton-Raphson method, such that

$\boldsymbol{\theta}_{n+1}=\boldsymbol{\theta}_{n}-\mathbf{H}^{-1}\boldsymbol{\nabla}$,

where, $\mathbf{H}$ is the hessian matrix, $\boldsymbol{\nabla}$ is the first order derivative vector, and$\boldsymbol{\theta}_{n}$ are parameter estimates in the $n$th iteration. From the maximum-likelihood parameter estimates, we calculate a z-statistic for each regression co-efficient, given by

$$z=\frac{\hat{\boldsymbol{\beta}}}{SE(\hat{\boldsymbol{\beta}})}$$

We then obtain a p-value for each regression parameter using

$$p=2\left( 1-\int_{-\infty}^{|z|} \frac{1}{\sqrt{2\pi}}e^{\frac{{-y}^{2}}{2}}dy \right)$$
